# Recurrent Somatic Chromosomal Abnormalities in Relapsed Extraocular Retinoblastoma

**DOI:** 10.3390/cancers13040673

**Published:** 2021-02-08

**Authors:** Rosario Aschero, Jasmine H. Francis, Daiana Ganiewich, Soledad Gomez-Gonzalez, Claudia Sampor, Santiago Zugbi, Daniela Ottaviani, Lauriane Lemelle, Marcela Mena, Ursula Winter, Genoveva Correa Llano, Gabriela Lamas, Fabiana Lubieniecki, Irene Szijan, Jaume Mora, Osvaldo Podhajcer, François Doz, François Radvanyi, David H. Abramson, Andrea S. Llera, Paula S. Schaiquevich, Cinzia Lavarino, Guillermo L. Chantada

**Affiliations:** 1 Pathology Service, Hospital de Pediatría JP Garrahan, Buenos Aires 1245, Argentina; rosarioaschero@gmail.com (R.A.); winter.u.a@gmail.com (U.W.); gslamas1@gmail.com (G.L.); flubieniecki@garrahan.gov.ar (F.L.); 2 National Scientific and Technical Research Council, CONICET, Buenos Aires 1425, Argentina; santiagozugbi@gmail.com (S.Z.); opodhajcer@leloir.org.ar (O.P.); allera@leloir.org.ar (A.S.L.); paulas@conicet.gov.ar (P.S.S.); 3 Ophthalmic Oncology Service, Memorial Sloan Kettering Cancer Center, New York, NY 10065, USA; francij1@mskcc.org (J.H.F.); Abramsod@mskcc.org (D.H.A.); 4 Laboratory of Molecular and Cellular Therapy, Instituto Leloir-Instituto de Investigaciones Bioquímicas de Buenos Aires (IIBBA), Buenos Aires 1405, Argentina; dganiewich@leloir.org.ar; 5 Developmental Tumor Biology Laboratory, Institut de Recerca Sant Joan de Déu, 08950 Barcelona, Spain; sgomezg@fsjd.org (S.G.-G.); jmora@sjdhospitalbarcelona.org (J.M.); clavarino@sjdhospitalbarcelona.org (C.L.); 6 Hematology-Oncology Service, Hospital de Pediatría JP Garrahan, Buenos Aires 1245, Argentina; claudiasampor@hotmail.com; 7 Innovative Treatments Unit, Hospital de Pediatría JP Garrahan, Buenos Aires 1245, Argentina; mafelcm@gmail.com; 8 University of Paris and Institut Curie (SIREDO Center: Care, Innovation and Reserach in pediatric, Adolescent and Young Adults Oncology), CNRS, UMR144, Equipe Labellisée Ligue Contre le Cancer, 75005 Paris, France; daniela.ottaviani@curie.fr (D.O.); lauriane.lemelle@curie.fr (L.L.); francois.doz@curie.fr (F.D.); francois.radvanyi@curie.fr (F.R.); 9 Pediatric Hematology and Oncology, Hospital Sant Joan de Déu, 08950 Barcelona, Spain; mcorreal@sjdhospitalbarcelona.org; 10 Genetic and Molecular Biology, University of Buenos Aires, Buenos Aires 1113, Argentina; iszijan@ffyb.uba.ar

**Keywords:** extraocular retinoblastoma, copy number alteration, genomic, metastasis, *BCOR* mutations

## Abstract

**Simple Summary:**

Relapse outside the eye of retinoblastoma (the most common eye cancer in children) is an uncommon event in developed countries, however it is the main cause of death in patients with retinoblastoma worldwide. The genomic features of this population are not known. We studied 23 cases from four countries and found a characteristic pattern in chromosomal copy number alterations that could help guide future clinical management of these patients.

**Abstract:**

Most reports about copy number alterations (CNA) in retinoblastoma relate to patients with intraocular disease and features of children with extraocular relapse remain unknown, so we aimed to describe the CNA in this population. We evaluated 23 patients and 27 specimens from 4 centers. Seventeen cases had extraocular relapse after initial enucleation and six cases after an initial preservation attempt. We performed an analysis of CNA and *BCOR* gene alteration by SNP array (Single Nucleotide Polymorfism array), whole-exome sequencing, IMPACT panel and CGH array (Array-based comparative genomic hybridization). All cases presented CNA at a higher prevalence than those reported in previously published studies for intraocular cases. CNA previously reported for intraocular retinoblastoma were found at a high frequency in our cohort: gains in 1q (69.5%), 2p (60.9%) and 6p (86.9%), and 16q loss (78.2%). Other, previously less-recognized, CNA were found including loss of 11q (34.8%), gain of 17q (56.5%), loss of 19q (30.4%) and *BCOR* alterations were present in 72.7% of our cases. A high number of CNA including 11q deletions, 17q gains, 19q loss, and *BCOR* alterations, are more common in extraocular retinoblastoma. Identification of these features may be correlated with a more aggressive tumor warranting consideration for patient management.

## 1. Introduction

Extraocular relapse of retinoblastoma, either after initial enucleation or an eye preservation attempt is rare in high-income countries, but it is the most common cause of disease-related death from this tumor [1,2]. Sites for extraocular relapse include the orbit, the central nervous system (CNS) and bone or bone marrow. Adjuvant treatment after enucleation, tailored by the presence of high-risk pathology factors (HRPF) is an effective way of preventing extraocular relapse in more than 95% of the initially enucleated cases [3,4,5]. Extraocular relapse is even less common in eyes that were conservatively treated with systemic, intra-arterial or intravitreal chemotherapy occurring in 1–2% of these cases, mostly after secondary enucleation [6]. Hence, despite being appropriately treated, there are still patients with retinoblastoma that experience a life-threatening extraocular relapse and there is little information about their biological features.

In recent years, copy number alterations (CNA) have been identified for risk assignment and translated to current clinical practice for some pediatric tumors [7,8]. However, the identification of the genomic features of retinoblastoma has focused mostly on intraocular cases and overall genomic features of a limited number of patients with extraocular relapse have been reported in the literature [9,10,11,12,13,14,15,16,17,18,19]. In such studies, other recurrent CNA besides the 13q loss or copy-neutral loss of heterozygosity (CN-LOH), such as gains in chromosomes 1q, 6p, 2p and losses in 16q have been identified in intraocular retinoblastoma, but none has been conclusively related to an increased risk of extraocular relapse. Gain of chromosome 6p, detected during conservative therapy, was identified as a risk factor for enucleation in studies using liquid biopsy from aqueous humor paracentesis [20]. However, despite them being more common in cases with HRPF, their association with extraocular relapse was not established. In addition, only a small percentage of patients present recurrent somatic mutations other than in the *RB1* gene such as in *BCL6 Corepressor* gene (*BCOR*) seen in 7 to 14% of cases and others which are even less common [10,11,16]. Hence, it is not known whether there are recurrent genomic abnormalities increasing the risk of extraocular relapse that could help in the identification of these higher risk patients before the occurrence of metastatic dissemination, or help interpret its tumorigenesis or mechanisms of tumor progression.

The relative rarity of extraocular relapse in retinoblastoma in high-income countries where genomic studies are available, limits the possibility of performing comprehensive studies in single centers, so collaborative studies involving less developed countries have the advantage of recruiting enough cases and generating more relevant information. Because of its rarity, usually only paraffin-embedded archival material is available for study and not infrequently, cases with extraocular relapse are not even biopsied because the extraocular relapse is diagnosed by cerebrospinal fluid (CSF) or bone marrow cytology. Overall, it is difficult to obtain tissue from metastatic sites. Furthermore, because of the poor prognosis of these patients, treatment is usually in the palliative care setting limiting further tissue availability for genomic studies. Hence, current next-generation sequencing studies reported for intraocular retinoblastoma or other pediatric malignancies are not available for extraocular retinoblastoma [11]. 

With the aim of describing genomic alterations of extraocular relapsed retinoblastoma, we assembled a multi-institutional cohort of patients and applied genomic techniques to examine CNA with the objective of identifying recurrent alterations that may help in the identification of a higher risk cohort and interpretation of their biology.

## 2. Results

### 2.1. Patients Characteristics and Treatment

Twenty-three patients with extraocular relapse of retinoblastoma were included (12 male, 11 female; 17 unilateral, 6 bilateral). The median age at diagnosis was 22 months (range 5 to 88), the median age at enucleation was 30.5 months (range 18 to 88) and the median age at extraocular relapse was 39 months (26 to 95). Extraocular relapse occurred after initial treatment with enucleation in 17 patients (group 1) and following secondary enucleation after failure of conservative therapy in 6 (group 2). 

In group 1, there were 14 patients with unilateral and three patients with bilateral disease. Ten patients received adjuvant therapy, five for postlaminar optic nerve invasion (pT3b), three for resection margin invasion (pT4) and one for massive choroidal invasion (pT3a). In the remaining case, neoadjuvant chemotherapy was given before planned enucleation because of massive buphthalmia which caused complete tumor necrosis in the enucleated eye, so it was not possible to assess the presence of HRPF or obtain genetic material from the primary tumor. Seven patients did not receive adjuvant therapy. For patients of this group, median age at diagnosis and at enucleation was 30 (18–88) and 31 (18–88) months, respectively, and median time from diagnosis to extraocular relapse was 7 months (range 2 to 30). Involved sites at extraocular relapse included the CNS in five patients, the bone or bone marrow in two, the orbit in three or a combination of them in seven cases. Treatment of extraocular relapse included intensive chemotherapy with (*n* = 6) or without (*n* = 9) autologous hematopoietic stem cell rescue and two palliative treatments. Six patients (35.3%) were alive and disease-free with a median follow-up of 92 months (range 31 to 277). 

For the six cases in group 2, there were three patients with bilateral retinoblastoma. Prior to enucleation, three patients had been treated with systemic and intra-arterial chemotherapy and three only with intra-arterial chemotherapy. In this group, one patient had postlaminar optic nerve invasion (pT3b) and one had scleral invasion (pT3c) and both received adjuvant therapy. Four patients did not receive adjuvant therapy. The median age at diagnosis was 17 months (range 5 to 22), and median time from diagnosis to extraocular relapse was 33.5 months (5–52). The median age at enucleation in this group was 30 months (25–60). Five patients had a systemic relapse (combined with orbital in one case and with CNS in one case), and one had orbit and CNS relapse. Treatment of extraocular relapse included intensive chemotherapy with (*n* = 4) or without (*n* = 2) autologous hematopoietic stem cell rescue. Three children (50%) were alive and disease-free with a median follow-up of 65 months (range 30 to 79). 

Overall, 14 (60.8%) patients died and 9 were alive and disease-free with a median follow-up of 79 months (range 30 to 277) (Table 1).

### 2.2. CNA Analysis

**Figure 1:**
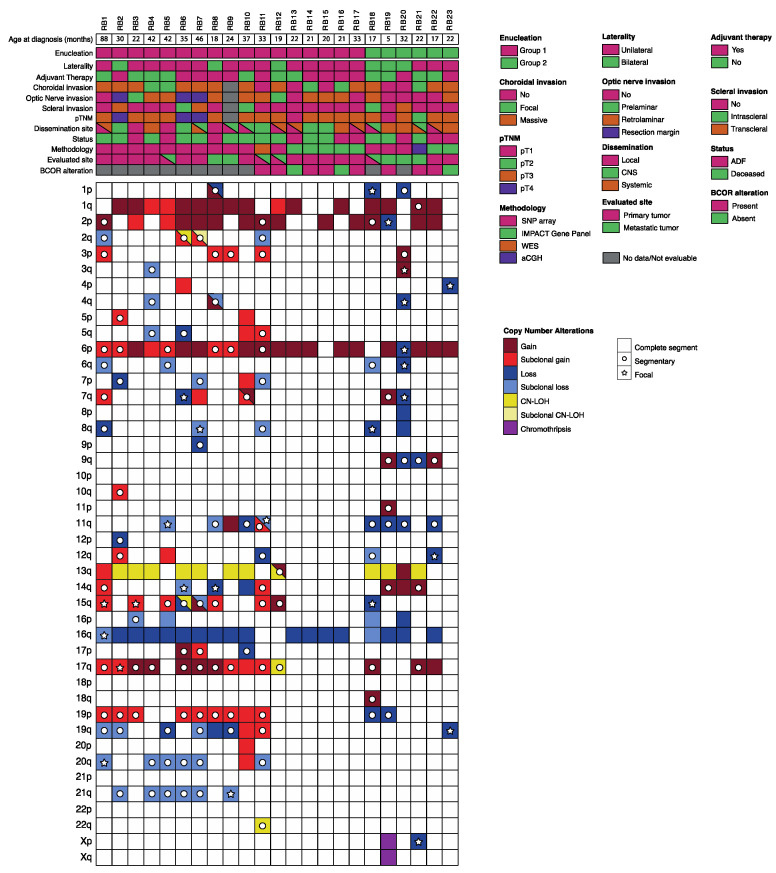
Clinical, pathologic, and genetic features of the 23 retinoblastoma patients. Each column represents an individual sample, and the rectangular boxes correspond to the status of each of the main characteristics depicted. Boxes are partitioned if more than one relevant feature coexists. For each clinical feature, the corresponding legend is represented on the left of the figure. For copy number alterations, gains are shown in red, blue represents losses, yellow is for CN-LOH and purple for chromothripsis, while the intensity of the color shade is proportional to the value of the log-ratio (LRR). In addition, full coloring means the copy number alteration comprises the whole segment (>90% of the arm), whereas a circle means part of the segment is altered and a star means it is a focal alteration (<15 genes). In cases where samples from primary site and metastatic site were available (patients RB5, RB11, RB12 and RB18), only the primary tumor results were represented. Abbreviations: CNS: central nervous system. WES: whole-exome sequencing. CN-LOH: copy neutral-loss of heterozygosity. ADF: alive disease free.

A total of 27 specimens were analyzed. Only the primary ocular tumor was available for genomic diagnosis in 14 patients, an extraocular specimen was available in 5 patients. Both the primary tumor and an extraocular specimen were available in the remaining four patients (Figure 1). The specimens from metastatic sites included orbital tissue in four cases, CSF cells in three, bone marrow cells in one and a lymph node biopsy in one case.

Common CNA, already reported for intraocular retinoblastoma such as 1q, 2p, 6p gains and 16q losses [11,12,14,15,16,17,18] were, respectively, detected in 69.5% (16/23), 60.9% (14/23), 86.9% (20/23), 78.2% (18/23) of patients (Figure 1). 

We found a high frequency of other novel CNA which were reported less frequently in intraocular retinoblastoma, such as 17q gains (56.5%, 13/23), 11q loss (34.8%, 8/23), 19q loss (30.4%, 7/23) and 21q loss (26.1%, 6/23). We compared the CNA distribution between the two groups (Figure 1). In group 1, we found that gains in 1q, 2p and 6p occurred in 70.6% (12/17), 64.7% (11/17) and 94.1% (16/17), respectively, while 16q loss occurred in 82.3% (14/17) of patients. In patients included in group 2, 50% (3/6) had gains in 2p, and 66.6% (4/6) showed 1q gains, 6p gains and 16q losses. These differences between groups were not significant. For the novel can, the prevalence of alterations in each group was also assessed. In group 1, the most frequent CNA were gain in 17q (58.8%), losses in 19q and 21q (35.5%) and loss in 11q (23.5%). For patients in group 2, the most frequent CNA was 11q loss (66.6%), followed by gain in 17q (50%) and only one case had loss in 19q (16.6%). No patient had alterations in 21q. These differences did not reach statistical significance.

These CNA, along with the recurrent 13q CN-LOH and others less frequently found, as well as the most relevant clinical features for each individual patient, are shown in Figure 1.

**Figure 2:**
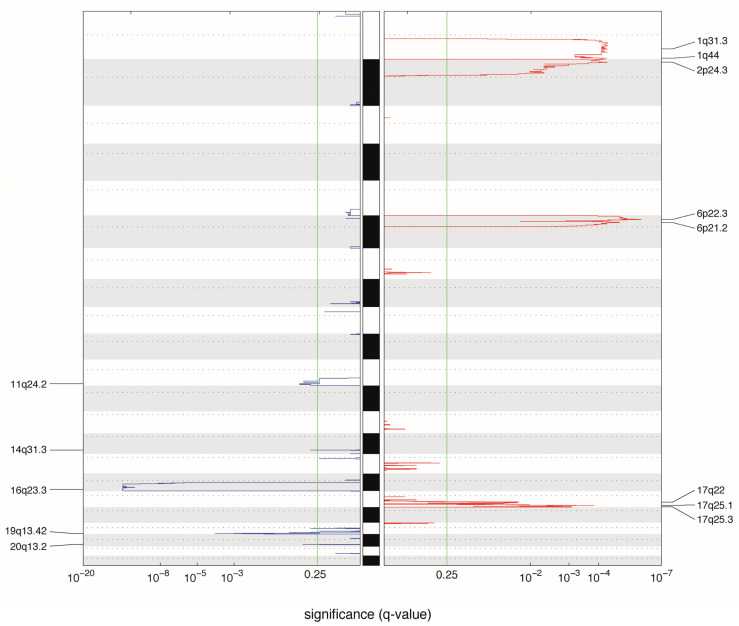
Significantly altered copy number regions identified by GISTIC. A karyogram overview of the cumulative copy number is shown separately for gains (red) and losses (blue) across the genome, depicted in the y-axis. In the x-axis, the GISTIC q-values are shown on a log scale and the green line represents the significance threshold (*q*-value = 0.25). Significantly altered regions are mentioned on the sides. For this analysis, samples from Hospital Garrahan and Sant Joan de Deu were used. In cases where samples from primary site and metastatic site were available (patients RB5, RB11, RB12 and RB18), only the primary tumor results were considered.

We performed a GISTIC analysis (Figure 2) to more accurately identify the frequently altered focal region on each chromosomal arm and to depict probable driver genes in several chromosomal regions. Previously reported features in intraocular patients that were also observed in our metastatic cases include genes associated to gains in 1q (including *MDM4* [17], *KIF14* [21] genes), 2p (*MYCN* [17]), and 6p (*DEK, E2F3*) [22] and 16q (*CDH11*) deletion [23]. The *ATM* tumor suppressor gene was significantly altered in cases with 11q deletion [24], which has not yet been described in retinoblastoma. 

Other potentially relevant CNA not reaching statistical significance were 5q34, 10p15 (*KLF6*), 17p13.3 (*TP53*)21q22 (*RUNX1*) deletions, and 7q31.33 (*MET* and *BRAF*) gain. These alterations are described in Table S1.

**Figure 3:**
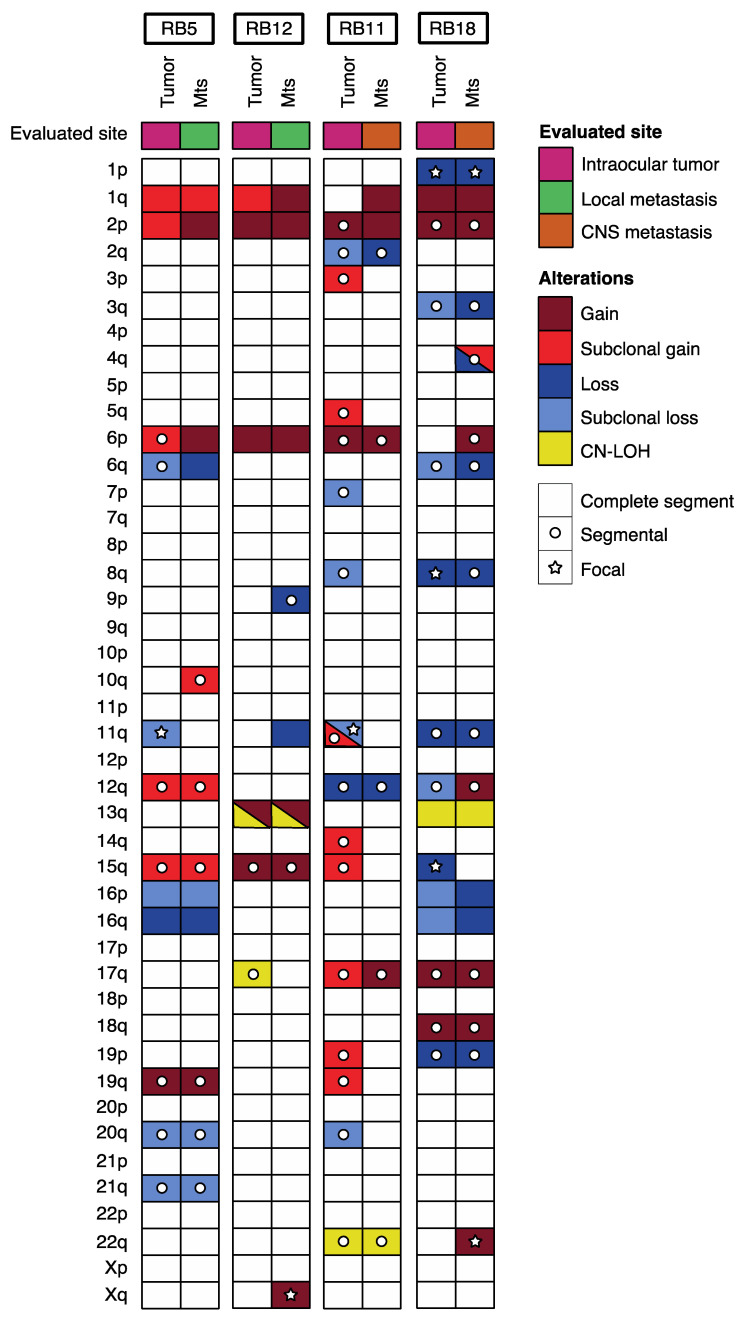
Landscape of somatic alterations in paired primary tumor metastatic site samples from retinoblastoma patients.

In four patients, we were able to study the CNA pattern in the primary tumor as well as in the metastatic sites. Three of them were primarily enucleated (group 1), while the remaining received conservative treatment prior to enucleation (group 2). CNA previously reported in intraocular retinoblastoma were detected in primary tumors of our patients and were also seen at the relapse with addition of new alterations, except in patients 11 and 12 in whom the 1q gain and 6p gain, respectively, appeared only at the metastatic site. Overall, we found ten alterations that were subclonal in primary tumor that have evolved to a clonal state at metastatic sites (Figure 3).

In addition, 11q loss was present at least in one specimen, in all patients included. In one case (patient 12), the alteration containing *ATM* was present in both the intraocular and the metastatic site. In patient 13 (previously reported as patient 1 by our group in another publication [25]), the orbital relapse showed an 11q loss spanning *ATM* that was absent in the primary tumor. Deletion of 11q not harboring the *ATM* gene region was observed subclonally and only in the primary tumors in two cases (patients 5 and 13, Figure 2). Gain of 17q was present in two cases both in primary and metastatic site (in one case evolving from a subclonal to clonal state).

Although 11q loss was found in 5 of 9 specimens analyzed from extraocular sites compared to 5 of 18 cases from intraocular tumors, this difference was not statistically significant (Table S2). 

We evaluated *BCOR* alterations in 11 samples: 7 by IMPACT, 3 by WES, and one by aCGH. Eight of the 11 (72.7%) samples showed alterations in *BCOR*. We were not able to distinguish an alteration pattern in this subgroup of patients since four of them had frameshift variants along the gene (two in exon 3, one in exon 9 and one in exon10), three had whole-gene deletion, and one had a truncating driver mutation. Other novel alterations recently reported in retinoblastoma such as mutations in *MGA, ARID1A, FAT1* and *ATRX* were not detected [11].

## 3. Discussion

In this report of the genomic alterations of a large cohort (considering its infrequency) of patients with relapsed extraocular retinoblastoma, we identified CNA at higher frequency than those reported for intraocular patients (Table 2). Retinoblastoma is thought to follow a multistep model for tumor progression [26,27] and bi-allelic inactivation of the *RB1* gene alone, though sufficient for retinoblastoma genesis, seems not sufficient for tumor progression, since all our patients show additional CNA. This high number of CNA, as an indicator of increasing genomic instability [26], confirms previous observations reporting CNA in 63% of children with HRPF and 22% in a recent series including non-enucleated eyes [11,20,27]. In addition, our data support a recent observation reporting that age at diagnosis is correlated with the number of CNA in non-metastatic patients with retinoblastoma regardless of laterality [27]. The median age at diagnosis of our patients with unilateral retinoblastoma having an extraocular relapse after initial enucleation was 33 months (range 20 to 88) which is substantially higher than that previously reported for unilateral disease (22 months in a South American report and 28 months in a recently reported series of patients with HRPF and CNA) [3,11] . Children with extraocular relapse occurring after initial enucleation may harbor an intrinsically more aggressive disease with faster progression compared to those who presented with less advanced disease in whom an ocular conservative approach was deemed feasible. Though not reaching statistical significance, in the former (group 1), unilateral disease predominated, and relapse tended to include the CNS in a higher proportion of cases. In the latter (group 2), bilateral cases predominated and tended to relapse later and less commonly in the CNS. In patients who had an extraocular relapse after failing conservative therapy, tumoral clones may emerge with time and treatment could affect their behavior as well.

One of the major findings of our study was the detection of novel recurrent CNA that were seldom reported in intraocular cases such as gains at chromosome 17q in 56.5%, deletion in chromosome 11q in 34.8%, loss of chromosome 19q in 30.4% and loss of 21q in 26.1% of the patients (Table 2). In addition, other CNA such as gains in chromosome 1q, 2p and 6p or losses at 16q which are typically found in patients with intraocular retinoblastoma were also detected in our cohort with an increased frequency compared to CNA reported for intraocular disease (Table 2) [11,12,14,15,16,17]. 

Even though it was reported that patients with HRPF have a higher number of chromosomal aberrations, none has been associated individually or in combination with an increased risk of metastatic relapse [11].

We confirmed previous reports showing common gain in chromosome 6p in high-risk patients shown in 87% of our cases with extraocular relapse, especially those primarily enucleated [20]. However, it has been reported in 61% of enucleated eyes without metastasis [20], thus the specificity as a predictor of metastasis appears limited. Gains in chromosome 2p involving *MYCN* were present in 60.9% of our patients, higher than the reported 43% for non-metastatic retinoblastoma with HRPF [11]. In addition, in our series, we excluded cases with *RB1^+/+^ MYCN^A^* which were included in other series [28,29], so the overall prevalence of *MYCN* alterations, considering only patients with *Rb1^−/−^* could be relatively higher. Interestingly, no patient in our cohort showed *MYCN^A^*. Chromosome 2p gains were more common in initially enucleated patients (group 1) suffering from an extraocular relapse, compared to those cases where extraocular relapse occurred after an initial preservation attempt (64.7% vs. 50%). Gains in 1q were evenly distributed (70.6% vs. 66.6%) in both groups. 

Even though these differences were not statistically significant, cases with gains in 2p—including *MYCN-* and gains in 1q likely represent more aggressive cases warranting initial enucleation. Loss of 1p was seen in three of our cases and it was previously reported in cases of extraocular retinoblastoma and it is associated to poor prognosis in neuroblastoma [19,30]. Nine patients (39.1%) did not harbor gains in chromosome 2p spanning *MYCN*. Two had 11q deletion which, as reported in neuroblastoma, may be mutually exclusive [31]; two had concomitant 17q gain and 19q loss; and two showed loss in 19q and 21q, respectively.

One of the most important findings in our study was the high prevalence of chromosome 11q deletions in our cohort of extraocular relapsed retinoblastoma. Chromosome 11q deletions have been only anecdotally found in intraocular retinoblastoma, but they are commonly described as a high-risk feature in 20 to 40% of neuroblastoma cases [31]. In neuroblastoma, it has been positively correlated with 4p loss and 7q gains which were seen in two of our patients as well [31]. In our series, all three patients with germline mutations in the *RB1* gene presenting with intraocular retinoblastoma followed by extraocular relapse after failing conservative therapy (group 2) had 11q deletion. As in neuroblastoma, haploinsufficiency of the *ATM* tumor suppressor gene may be a candidate driver gene for 11q region loss [24]. 

We found a 56.5% frequency of gains in chromosome 17q in our series. The highest prevalence of 17q gains in retinoblastoma was recently reported as 12.5% in patients with HRPF [11]. One of the four cases with 17q gain in that series had metastatic disease, so the figure for non-metastastic HRPF patients is even lower [11]. Alterations in chromosome 17q have been reported in 3 of 14 *RB1^+/+^MYCN^A^* patients with no extraocular disease [28]. Chromosome 17q gain has been reported in other tumors especially in neuroblastoma and also in medulloblastoma [32,33]. In our series, GISTIC analysis, was not able to find a candidate driver gene for this alteration.

Chromosome 19q loss is another uncommonly reported CNA in retinoblastoma but it occurred in 30.4% of our patients. Previously, it had been reported in two patients who developed metastatic disease (one with concomitant 11q loss and 17q gain) [11] . In neuroblastoma, it has been reported in association with a higher risk of local relapse and it may be detected only when biological material is evaluated at relapse [34]. In two of our cases, 19q deletions were seen in cases with an orbital relapse which would parallel the findings described in neuroblastoma. 

There were other CNA in smaller proportions and not significantly found by GISTIC that were more common in our series that might also be risk factors for extraocular relapse such as the previously unreported 21q22 deletion containing *RUNX1*. Additionally, the deletion of 5q was seen in two of our patients, which has been previously reported in two patients with metastatic retinoblastoma [14]. The potential importance of these alterations in patients with metastatic retinoblastoma requires further study.

We also analyzed paired samples including the primarily enucleated eye and the metastatic site in four patients. As in neuroblastoma [31], specimens obtained from a relapsed site had a higher number of CNA compared to the primary tumor [35] (and 10 alterations that were found in a subclonal population were seen clonally in the relapse specimen, suggesting clonal evolution. In one case, the 11q deletion was present in both primary tumor and metastatic sites. One patient (RB12), previously reported by our group, showed 11q deletion only in the metastatic site [25]. This phenomenon was also reported for neuroblastoma where it has been reported that 11q deletion may be present only in the relapsed specimen, suggesting clonal evolution at the metastatic site or a subclonal population not detectable in the primary tumor that had the ability to metastasize [31]. In the two remaining cases, a subclonal 11q deletion was detected in the primary tumor and it was not detectable in the metastatic relapse. However, in general, 11q losses were more common in extraocular specimens. These findings and the presence of subclonal alterations seen in many of our cases, suggest intra-tumoral heterogeneity as it was previously described for retinoblastoma and other tumors with subclones capable to metastasize and may acquire additional genomic alterations or lose others at the extraocular site [25,35]. Deeper and single-cell sequencing studies should describe this phenomenon with greater precision.

As in other tumors with segmental chromosomal abnormalities likes neuroblastoma, the evaluation of the minimal region of loss or gain to identify putative candidate genes associated to increased tumor aggressiveness did not yield conclusive results in retinoblastoma [9,17]. From our data, it cannot be ascertained whether these segmental alterations have a direct role in tumor progression or if they are only surrogate markers of other genomic abnormalities. Alterations in the *ATM* gene have been identified in patients with 11q deletions and their role in metastasis should be explored. In other tumors such as neuroblastoma, it has been debated if the CNA, leading to genomic instability, represent the driver for tumor progression and aggressiveness [26,35].

The elucidation of the precise molecular mechanisms underlying segmental alterations in retinoblastoma and the role of additional somatic mutations such as those in *BCOR* or others would be helpful for the interpretation of our findings. Alterations in *BCOR* could only be evaluated in 11 patients due to lack of material but they were present in 72.7% of our cases which is much higher than the reported 7–14% prevalence in intraocular cases [11,16]. In a recent study, *BCOR* mutations were seen more commonly in unilateral intraocular cases with HRPF [11]. In our series, it was present also in patients with germline mutations and extraocular relapses, however the number of patients studied is small.

Taking our data together, we hypothesize that tumors diagnosed in older children accumulate genomic alterations over time making cancer cells more prone to acquire additional high-risk features leading to greater genomic instability resulting in tumor progression. This would explain the poorer prognosis of children with retinoblastoma in less-favored settings where delayed diagnosis is common. Conversely, in patients with less advanced disease at diagnosis and when managed conservatively, subclones harboring 11q abnormalities and other CNA may develop explaining the extraocular relapses.

This study has many limitations. Even though it is the largest genomic analysis of extraocular retinoblastoma reported to date, it represents a small cohort of mostly archival cases. Additionally, different technologies were used which may have not been able to capture all the important features. This is a retrospective analysis mostly dependent on the availability of biological material for genomic studies and does not represent a consecutive patient cohort. In five patients, only a metastatic site was available, so it was not possible to evaluate the CNA in the enucleated eye. In addition, it was not possible, due to the low number of patients in each category to correlate the CNA with a specific relapse pattern with statistical confidence. A prospective study correlating the presence of HRPF with CNA and outcome should confirm these observations. As proposed for other pediatric tumors, CNA may also be detected by the evaluation of circulating free tumor DNA [36,37]. 

## 4. Materials and Methods 

### 4.1. Patient Samples

Patients with intraocular retinoblastoma who had extraocular relapse either after initial treatment with enucleation or after failure of conservative therapy were included. Availability of biological material with appropriate informed consents for tissue disposition was considered an inclusion criterion. Extraocular relapse was defined as disease involving the orbit, CNS and/or systemic (e.g., bone and bone marrow) dissemination. Patients with trilateral retinoblastoma and those with *RB1^+/+^ MYCN^A^* were excluded. 

Patients from the following institutions were included: Hospital JP Garrahan (Buenos Aires, Argentina) (*n* = 14), Memorial Sloan Kettering Cancer Center (New York, NY, USA) (*n* = 7), Institut Curie (Paris, France) (*n* = 1) and Sant Joan de Déu (Barcelona, Spain) (*n* = 1). 

At the Hospital JP Garrahan, all patients with extraocular relapse, diagnosed from 2015 to 2019 were enrolled into a prospective study for biological specimen procurement including collection of fresh tumor material as per international guidelines [34], for DNA collection for genomic analysis, cell culture in tumorspheres and patient-derived xenografts. In these patients, whole-exome sequencing of the tumor was performed in parallel with the study of germline mutations of the *RB1* gene. 

For those from whom there was no fresh tumor collected, archival paraffin embedded tissue (FFPE) from the primary tumor and/or extraocular site (if available) was retrieved and tumor DNA was extracted by commercial affinity columns (PureLink™ Genomic DNA Mini Kit, Thermo Fisher, Waltham, MA 02451, USA).

### 4.2. Chromosomal Copy Number Alteration Analysis

CNA were analyzed using different techniques according to biological samples available. OncoScan™ CNV Assay (Thermo Fisher Scientific, Waltham, MA, USA) was used for FFPE samples (*n* = 14) while CytoScan HD (Thermo Fisher Scientific, Waltham, MA, USA, *n* = 2) or whole-exome sequencing (WES, *n* = 3) was used for frozen tissue when available. Comparative Genomic Hybridization array (Agilent Technologies, Santa Clara, CA, USA, *n* = 1) was used for patient from Institut Curie and IMPACT panel for patients treated at Memorial Sloan Kettering (*n* = 7).

The OncoScan and CytoScan hybridization was done at Institut Hospital del Mar d´Investigacion Médiques (IMIM), (Barcelona, Spain) following Affymetrix instructions. The data were analyzed at Hospital Sant Joan de Déu with Chromosome Analysis Suite software v3.0 (ChAS) (Affymetrix, Inc., Waltham, MA 02451, USA ) applying the parameters depicted by the manufacturer to determine the chromosomic alterations (gain, loss, or LOH). Exome capture was performed by SureSelect V7 (Agilent Technologies, Santa Clara, CA, USA) and sequenced in a Novaseq 6000 (Illumina, San Diego, CA, USA) followed by the bioinformatic analysis as previously reported [34]. Both results were manually reviewed. The aCGH assay was performed at Institut Curie, (Paris, France) using 180K CGH/LOH custom chip and Cytogenomics V2.9.2.4 software (Agilent, Santa Clara, CA 95051, USA). The MSK-IMPACT is a panel for next generation sequencing reporting that is performed routinely in patients treated at MSKCC [38]. It is capable of detecting a variety of mutations, gene amplifications and deletions, and genomic rearrangements and tumor mutation burden [38]. We evaluated alterations present in a broad or focal fashion and clonal or subclonal state. In all cases, alterations were manually classified into complete segment if the alteration comprised more than 90% of the arm, focal if 15 or less genes were implicated in the region and segmentary for any alteration in between. 

Analysis of significant CNAs was performed using GISTIC v2.0.23 with default parameters and confidence level of 0.9. Events were considered as clonal when the absolute value of LRR was greater than 0.5. Given the relatively small sample size, other non-significant regions (*q*-value > 0.25) containing cancer-related genes were identified when re-running GISTIC using 0.8 as the new q-value threshold and all GISTIC results were manually reviewed. Candidate driver genes for each region were obtained by consulting the COSMIC Cancer Gene Census (accessed 14 January 2020). [39] All samples from Garrahan and Sant Joan de Déu Hospitals were included in the analysis, except in the case of paired samples, in which only the intraocular tumor was considered.

### 4.3. Clinical Data

Clinical data were retrieved from the institutional databases. Invasion of the optic nerve, the choroid or the sclera were defined as per the International Retinoblastoma Staging System [40] and all cases were re-classified according to the TNMH classification. Data were gathered into a single database and cases were de-identified at the participating institutions. Treatment of extraocular relapse was decided independently in each institution.

### 4.4. Statistical Analysis

Statistical analysis was performed in GraphPad Prism Version 8.0.1 (GraphPad Software. San Diego, CA, USA). Comparison of clinical and histologic features were performed using the Mann–Whitney test for continuous variables (diagnosis age, relapse age) and the Fisher’s exact test for categorical variables (all other variables). A *p*-value cutoff of 0.05 was used to assess statistical significance.

## 5. Conclusions

Patients with extraocular relapse of retinoblastoma show a high number of CNA and tend to be older at diagnosis than those who do not relapse. Several novel non-random CNA, including some not seen in intraocular disease such as 17q gains, 11q loss and 19q loss were identified in more than 30% of the cases and they may be related to increased tumor aggressiveness. Thereby, this study potentially provides opportunities to improve clinical care by identifying a genomic signature to enhance the detection of retinoblastoma with a high risk of relapse which should be confirmed in a prospective study.

## Figures and Tables

**Table 1:** Clinical and histopathological features of patients.

Patients Features	Group 1	Group 2	*p*	Test
*n* = 23	17	6		
Laterality				
Unilateral	14	3	0.3121	Chi square
Bilateral	3	3		
Age at diagnosis (months)				
Median	30	17	0.0041	Mann Whitney
Range	18–88	5–22		
Age at relapse (months)				
Median	39	47.5	0.4205	Mann Whitney
Range	26–95	27–67		
HRPF				
No HRPF	1	2		
Massive choroidal invasion alone	4	1		
PLONI +/− choroidal/scleral invasion	8	2		
Scleral invasion no PLONI	0	1		
Resection margin of the optic nerve invasion	3	0		
Not evaluable	1	0		
Adjuvant therapy				
None	7	4	0.3707	Fisher´s exact
Yes	10	2		
Sites of extraocular relapse				
Isolated orbit	3	0		
Orbit + CNS	2	1		
Orbit + Systemic	4	1		
Systemic	2	3		
Isolated CNS	5	0		
Systemic + CNS	1	1		
Treatment of extraocular relapse				
Systemic chemotherapy +/− radiotherapy no ASCR	9	2		
Systemic chemotherapy +/− radiotherapy + ASCR	6	4		
Palliative treatment	2	0		
Outcome				
Death	11	3	0.643	Fisher´s exact
Alive disease-free	6	3		

Group 1: initial enucleation. Group 2, secondary enucleation after initial preservation attempt. Abbreviations: HRPF: high-risk pathology factors; PLONI: postlaminar optic nerve invasion; CNS: central nervous system; ASCR: autologous hematopoietic stem cell rescue.

**Table 2:** Comparison of reported copy number alterations (CNA) (besides *RB1*) in series with mostly intraocular cases and this study.

CNA	This Series	Overall	Mairal et al., 2000 [18]	Chen et al., 2001 [17]	Lillington et al., 2003 [14]	Zielinski et al., 2005 [15]	Sampieri et al., 2009 [12]	Kooi et al., 2016 [16]	Afshar et al., 2019 [11]
*n*	23	230	24	50	49	17	18	71	28 *
Techniques	Various genomic techniques	Various genomic techniques	Karyotype and CGH	CGH	CGH	CGH	CGH	WES	NGS (UCSF500 Cancer Panel)
Any CNA	100%	87%	96%	82%	96%	88%	67%	83%	96%
Gain 1q	70%	53%	50%	56%	57%	71%	22%	54%	64%
Gain 2p	61%	36%	38%	34%	43%	29%	22%	41%	43%
Gain 6p	87%	57%	54%	46%	69%	59%	39%	68%	61%
Loss 16q	78%	37%	46%	14%	39%	41%	11%	45%	61%
Loss 11q	35%	4%	NF	NF	NF	NF	11%	NF	14%
Gain 17q	56%	5%	13%	12%	NF	NF	NF	NF	7%
Loss 19q	30%	1%	NF	NF	NF	NF	NF	NF	7%
Other	Loss 21q (26%)				Gain 19q (27%)	Gain 19q (24%)	Gain 9q (17%)		
BCOR	73% (8/11)	12%	NF	NF	NF	NF	NF	10%	14%

* Calculated only on *RB1^−/−^* cases. Abbreviations: CGH: comparative genomic hybridization array; WES: whole-exome sequencing; NGS: next generation sequencing; NF: not found.

## Data Availability

The data presented in this study are available on request from the corresponding author.

## Supplementary Materials

The following are available online at https://www.mdpi.com/2072-6694/13/4/673/s1, Table S1: Regions identified by GISTIC as significantly altered in our cohort, Table S2: Comparison between CNA profiles in intraocular and extraocular tumors.
